# On Geometry of Information Flow for Causal Inference

**DOI:** 10.3390/e22040396

**Published:** 2020-03-30

**Authors:** Sudam Surasinghe, Erik M. Bollt

**Affiliations:** 1 Department of Mathematics, Clarkson University, Potsdam, NY 13699, USA; 2 Department of Electrical and Computer Engineering, Clarkson Center for Complex Systems Science (C3S2), Clarkson University, Potsdam, NY 13699, USA

**Keywords:** causal inference, transfer entropy, differential entropy, correlation dimension, Pinsker’s inequality, Frobenius–Perron operator

## Abstract

Causal inference is perhaps one of the most fundamental concepts in science, beginning originally from the works of some of the ancient philosophers, through today, but also weaved strongly in current work from statisticians, machine learning experts, and scientists from many other fields. This paper takes the perspective of information flow, which includes the Nobel prize winning work on Granger-causality, and the recently highly popular transfer entropy, these being probabilistic in nature. Our main contribution will be to develop analysis tools that will allow a geometric interpretation of information flow as a causal inference indicated by positive transfer entropy. We will describe the effective dimensionality of an underlying manifold as projected into the outcome space that summarizes information flow. Therefore, contrasting the probabilistic and geometric perspectives, we will introduce a new measure of causal inference based on the fractal correlation dimension conditionally applied to competing explanations of future forecasts, which we will write $GeoC_{y\to x}$. This avoids some of the boundedness issues that we show exist for the transfer entropy, $T_{y\to x}$. We will highlight our discussions with data developed from synthetic models of successively more complex nature: these include the Hénon map example, and finally a real physiological example relating breathing and heart rate function.

## 1. Introduction

Causation Inference is perhaps one of the most fundamental concepts in science, underlying questions such as “what are the causes of changes in observed variables”. Identifying, indeed even defining causal variables of a given observed variable is not an easy task, and these questions date back to the Greeks [1,2]. This includes important contributions from more recent luminaries such as Russel [3], and from philosophy, mathematics, probability, information theory, and computer science. We have written that [4], “a basic question when defining the concept of information flow is to contrast versions of reality for a dynamical system. Either a subcomponent is closed or alternatively there is an outside influence due to another component”. Claude Granger’s Nobel prize [5] winning work leading to Granger Causality (see also Wiener [6]) formulates causal inference as a concept of quality of forecasts. That is, we ask, does system *X* provide sufficient information regarding forecasts of future states of system *X* or are there improved forecasts with observations from system *Y*? We declare that *X* is not closed, as it is receiving influence (or information) from system *Y*, when data from *Y* improve forecasts of *X*. Such a reduction of uncertainty perspective of causal inference is not identical to the interventionists’ concept of allowing perturbations and experiments to decide what changes indicate influences. This data oriented philosophy of causal inference is especially appropriate when (1) the system is a dynamical system of some form producing data streams in time, and (2) a score of influence may be needed. In particular, contrasting forecasts is the defining concept underlying Granger Causality (G-causality), and it is closely related to the concept of information flow as defined by transfer entropy [7,8], which can be proved as a nonlinear version of Granger’s otherwise linear (ARMA) test [9]. In this spirit, we find methods such as Convergent Cross-Mapping method (CCM) [10], and causation entropy (CSE) [11] to disambiguate direct versus indirect influences [11,12,13,14,15,16,17,18]. On the other hand, closely related to information flow are concepts of counter factuals: “what would happen if...” [19] that are foundational questions for another school leading to the highly successful Pearl “Do-Calculus” built on a specialized variation of Bayesian analysis [20]. These are especially relevant for nondynamical questions (inputs and outputs occur once across populations), such as a typical question of the sort, “why did I get fat” may be premised on inferring probabilities of removing influences of saturated fats and chocolates. However, with concepts of counter-factual analysis in mind, one may argue that Granger is less descriptive of causation inference, but rather more descriptive of information flow. In fact, there is a link between the two notions for so-called “settable" systems under a conditional form of exogeneity [21,22].

**Figure 1:**
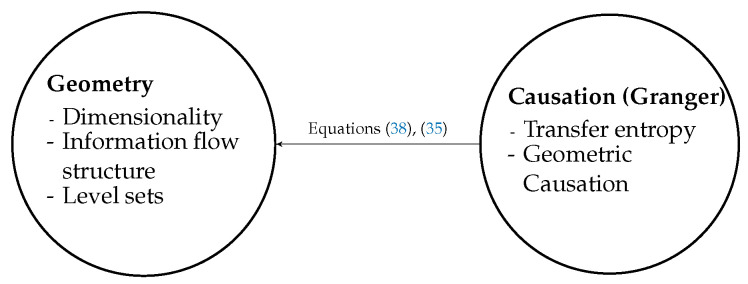
Summary of the paper and relationship of causation and geometry.

This paper focuses on the information flow perspective, which is causation as it relates to G-causality. The role of this paper is to highlight connections between the probabilistic aspects of information flow, such as Granger causality and transfer entropy, to a less often discussed geometric picture that may underlie the information flow. To this purpose, here we develop both analysis and data driven concepts to serve in bridging what have otherwise been separate philosophies. Figure 1 illustrates the two nodes that we tackle here: causal inference and geometry. In the diagram, the equations that are most central in serving to bridge the main concepts are highlighted, and the main role of this paper then could be described as building these bridges.

When data are derived from a stochastic or deterministic dynamical system, one should also be able to understand the connections between variables in geometric terms. The traditional narrative of information flow is in terms of comparing stochastic processes in probabilistic terms. However, the role of this paper is to offer a unifying description for interpreting geometric formulations of causation together with traditional statistical or information theoretic interpretations. Thus, we will try to provide a bridge between concepts of causality as information flow to the underlying geometry since geometry is perhaps a natural place to describe a dynamical system.

Our work herein comes in two parts. First, we analyze connections between information flow by transfer entropy to geometric quantities that describe the orientation of underlying functions of a corresponding dynamical system. In the course of this analysis, we have needed to develop a new “asymmetric transfer operator” (asymmetric Frobenius–Perron operator) evolving ensemble densities of initial conditions between spaces whose dimensionalities do not match. With this, we proceed to give a new exact formula for transfer entropy, and from there we are able to relate this Kullback–Leibler divergence based measure directly to other more geometrically relevant divergences, specifically total variation divergence and Hellinger divergence, by Pinsker’s inequality. This leads to a succinct upper bound of the transfer entropy by quantities related to a more geometric description of the underlying dynamical system. In the second part of this work, we present numerical interpretations of transfer entropy $TE_{y\to x}$ in the setting of a succession of simple dynamical systems, with specifically designed underlying densities, and eventually we include a heart rate versus breathing rate data set. Then, we present a new measure in the spirit of G-causality that is more directly motivated by geometry. This measure, $GeoC_{y\to x}$, is developed in terms of the classical fractal dimension concept of correlation dimension.

In summary, the main theme of this work is to provide connections between probabilistic interpretations and geometric interpretations of causal inference. The main connections and corresponding sections of this paper are summarized as a dichotomy: Geometry and Causation (information flow structure) as described in Figure 1. Our contribution in this paper is as follows:

- In traditional methods, causality is estimated by probabilistic terms. In this study, we present analytical and data driven approach to identify causality by geometric methods, and thus also a unifying perspective.
- We show that a derivative (if it exists) of the underlining function of the time series has a close relationship to the transfer entropy (Section 2.3).
- We provide a new tool called geoC to identify the causality by geometric terms (Section 3).
- Correlation dimension can be used as a measurement for dynamics of a dynamical system. We will show that this measurement can be used to identify the causality (Section 3).

Part I: Analysis of Connections between Probabilistic Methods and Geometric Interpretations

## 2. The Problem Setup

For now, we assume that x,y are real valued scalars, but the multi-variate scenario will be discussed subsequently. We use a shorthand notation, $x:=x_{n}$, $x^{\prime }:=x_{n+1}$ for any particular time *n*, where the prime (${}^{\prime }$) notation denotes “next iterate”. Likewise, let z=(x,y) denote the composite variable, and its future composite state, $z^{\prime }$. Consider the simplest of cases, where there are two coupled dynamical systems written as discrete time maps,

(1) $$\begin{array}{ccc} x^{\prime } & = & f_{1}\left(x,y\right), \end{array}$$

(2) $$\begin{array}{ccc} y^{\prime } & = & f_{2}\left(x,y\right). \end{array}$$

The definition of transfer entropy [7,8,23], measuring the influence of coupling from variables *y* onto the future of the variables *x*, denoted by $x^{\prime }$ is given by:

(3) $$T_{y\to x}=D_{KL}(p\left(x^{\prime }|x\right)||p\left(x^{\prime }|x,y\right)).$$

This hinges on the contrast between two alternative versions of the possible origins of $x^{\prime }$ and is premised on deciding one of the following two cases: Either

(4) $$x^{\prime }=f_{1}\left(x\right),\;\;\mathrm{or}\;\;x^{\prime }=f_{1}\left(x,y\right)$$

is descriptive of the actual function $f_{1}$. The definition of $T_{y\to x}$ is defined to decide this question by comparing the deviation from a proposed Markov property,

(5) $$p\left(x^{\prime }|x\right)\overset{?}{=}p\left(x^{\prime }|x,y\right).$$

The Kullback–Leibler divergence used here contrasts these two possible explanations of the process generating $x^{\prime }$. Since $D_{KL}$ may be written in terms of mutual information, the units are as any entropy, bits per time step. Notice that we have overloaded the notation writing $p(x^{\prime }|x)$ and $p(x^{\prime }|x,y)$. Our practice will be to rely on the arguments to distinguish functions as otherwise different (likewise distinguishing cases of $f_{1}\left(x\right)$ versus $f_{1}\left(x,y\right)$.

**Figure 2:**
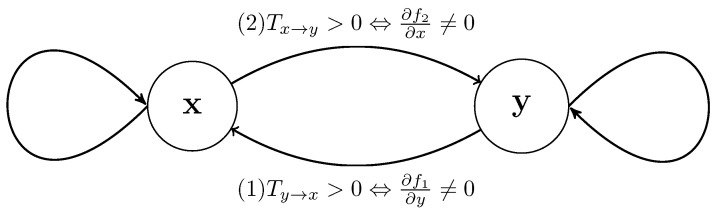
A directed graph presentation of the coupling stucture questions corresponding to Equations (1) and (2).

Consider that the coupling structure between variables may be characterized by the directed graph illustrated in Figure 2.

In one time step, without loss of generality, we may decide on Equation (4), the role of *y* on $x^{\prime }$, based on $T_{y\to x}>0$, exclusively in terms of the details of the argument structure of $f_{1}$. This is separate from the reverse question of $f_{2}$ as to whether $T_{x\to y}>0$. In geometric terms, assuming $f_{1}\in C^{1}\left(\Omega_{1}\right)$, it is clear that, unless the partial derivative $\frac{\partial f_{1}}{\partial y}$ is zero everywhere, then the *y* argument in $f_{1}\left(x,y\right)$ is relevant. This is not a necessary condition for $T_{y\to x}>0$, which is a probabilistic statement, and almost everywhere is sufficient.

### 2.1. In Geometric Terms

**Figure 3:**
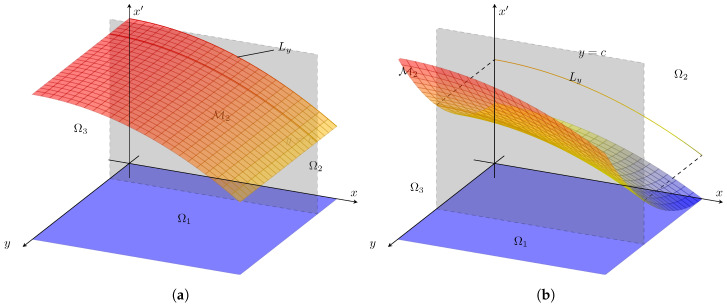
Ω_2_ = *X* × *X*′ manifold and *L_y_* level set for (**a**) *x*′ = *f*_1_(*x*) = −0.005*x*^2^ + 100, (**b**) *x*′ = *f*_1_(*x*, *y*) = −0.005*x*^2^ + 0.01*y*^2^ + 50. The dimension of the projected set of (*x*, *x*′) depends on the causality as just described. Compare to Figure 4 and Equation (27).

Consider a manifold of points $\left(x,y,x^{\prime }\right)\in X\times Y\times X^{\prime }$ as the graph over $\Omega_{1}$, which we label $M_{2}$. In the following, we assume $f_{1}\in C^{1}\left(\Omega_{1}\right),\Omega_{1}\subset X\times Y$. Our primary assertion here is that the geometric aspects of the set $\left(x,y,x^{\prime }\right)$ projected into $\left(x,x^{\prime }\right)$ distinguishes the information flow structure. Refer to Figure 3 for notation. Let the level set for a given fixed *y* be defined,

(6) $$L_{y}:=\left\{\left(x,x^{\prime }\right):x^{\prime }=f\left(x,y\right),y=constant\right\}\in \Omega_{2}=X\times X^{\prime }$$

When these level sets are distinct, then the question of the relevance of *y* to the outcome of $x^{\prime }$ is clear:

- If $\frac{\partial f_{1}}{\partial y}=0$ for all $\left(x,y\right)\in \Omega_{1}$, then $L_{y}=L_{\tilde{y}}$ for all $y,\tilde{y}$.

Notice that, if the *y* argument is not relevant as described above, then $x^{\prime }=f_{1}\left(x\right)$ better describes the associations, but if we nonetheless insist to write $x^{\prime }=f_{1}\left(x,y\right)$, then $\frac{\partial f_{1}}{\partial y}=0$ for all $\left(x,y\right)\in \Omega_{1}$. The converse is interesting to state explicitly,

- If $L_{y}\neq L_{\tilde{y}}$ for some $y,\tilde{y}$, then $\frac{\partial f_{1}}{\partial y}\neq 0$ for some $\left(x,y\right)\in \Omega_{1}$, and then $x^{\prime }=f_{1}\left(x\right)$ is not a sufficient description of what should really be written $x^{\prime }=f_{1}\left(x,y\right)$. We have assumed $f_{1}\in C^{1}\left(\Omega_{1}\right)$ throughout.

### 2.2. In Probabilistic Terms

Considering the evolution of *x* as a stochastic process [8,24], we may write a probability density function in terms of all those variables that may be relevant, $p(x,y,x^{\prime })$. Contrasting the role of the various input variables requires us to develop a new singular transfer operator between domains that do not necessarily have the same number of variables. Notice that the definition of transfer entropy (Equation (3)) seems to rely on the absolute continuity of the joint probability density $p(x,y,x^{\prime })$. However, that joint distribution of p(x,y,f(x,y)) is generally not absolutely continuous, noticing its support is $\left\{\left(x,y,f\left(x,y\right)\right):\left(x,y\right)\in \Omega_{x}\times \Omega_{y}\subseteq R^{2}\right\}$, a measure 0 subset of $R^{3}$. Therefore, the expression h(f(X,Y)|X,Y) is not well defined as a differential entropy and hence there is a problem with transfer entropy. We expand upon this important detail in the upcoming subsection. To guarantee existence, we interpret these quantities by convolution to smooth the problem. Adding an “artificial noise” with standard deviation parameter ϵ allows definition of the conditional entropy at the singular limit ϵ approaches to zero, and likewise the transfer entropy follows.

The probability density function of the sum of two continuous random variables (U,Z) can be obtained by convolution, $P_{U+Z}=P_{U}*P_{Z}$. Random noise (*Z* with mean E(Z)=0 and variance $V\left(Z\right)=C\epsilon^{2}$) added to the original observable variables regularizes, and we are interested in the singular limit, ϵ→0. We assume that *Z* is independent of X,Y. In experimental data from practical problems, we argue that some noise, perhaps even if small, is always present. Additionally, noise is assumed to be uniform or normally distributed in practical applications. Therefore, for simplicity of the discussion, we mostly focused on those two distributions. With this concept, Transfer Entropy can now be calculated by using $h(X^{\prime }|X,Y)$ and $h(X^{\prime }|X)$ when

(7) $$\begin{array}{c} X^{\prime }=f\left(X,Y\right)+Z, \end{array}$$

where now we assume that X,Y,Z∈R are independent random variables and we assume that $f:\Omega_{x}\times \Omega_{y}\to R$ is a component-wise monotonic (we will consider the monotonically increasing case for consistent explanations, but one can use monotonically decreasing functions in similar manner) continuous function of X,Y and $\Omega_{x},\Omega_{y}\subseteq R$.

#### Relative Entropy for a Function of Random Variables

Calculation of transfer entropy depends on the conditional probability. Hence, we will first focus on conditional probability. Since for any particular values x,y the function value f(x,y) is fixed, we conclude that $X^{\prime }|x,y$ is just a linear function of *Z*. We see that

(8) $$\begin{array}{c} p_{X^{\prime }|X,Y}\left(x^{\prime }|x,y\right)=Pr\left(Z=x^{\prime }-f\left(x,y\right)\right)=p_{Z}\left(x^{\prime }-f\left(x,y\right)\right), \end{array}$$

where $p_{Z}$ is the probability density function of *Z*.

Note that the random variable $X^{\prime }|x$ is a function of (Y,Z). To write U+Z, let U=f(x,Y). Therefore, convolution of densities of *U* and *Z* gives the density function for $p(x^{\prime }|x)$ (See Section 4.1 for examples). Notice that a given value of the random variable, say X=α, is a parameter in *U*. Therefore, we will denote U=f(Y;α). We will first focus on the probability density function of *U*, $p_{U}\left(u\right)$, using the Frobenius–Perron operator,

(9) $$\begin{array}{c} p_{U}\left(u\right)=\sum_{y:u=f(y;\alpha )}\frac{p_{Y}\left(f\left(y;\alpha \right)\right)}{|f^{\prime }\left(f\left(y;\alpha \right)\right)|}. \end{array}$$

In the multivariate setting, the formula is extended similarly interpreting the derivative as the Jacobian matrix, and the absolute value is interpreted as the absolute value of the determinant. Denote $Y=\left(Y_{1},\;Y_{2},\;\cdots ,\;Y_{n}\right),\;g\left(Y;\alpha \right)=\left(g_{1},\;g_{2},\;\cdots ,\;g_{n}\right)$ and $U=f\left(\alpha ,Y\right):=g_{1}\left(Y;\alpha \right)$; and the vector $V=\left(V_{1},\;V_{2},\;\cdots ,\;V_{n-1}\right)\in R^{n-1}$ such that $V_{i}=g_{i+1}\left(Y\right):=Y_{i+1}$ for i=1,2,⋯,n−1. Then, the absolute value of the determinate of the Jacobian matrix is given by: $|J_{g}\left(y\right)|=|\frac{\partial g_{1}\left(y;\alpha \right)}{\partial y_{1}}|$. As an aside, note that *J* is lower triangular with diagonal entries $d_{ii}=1$ for i>1. The probability density function of *U* is given by

(10) $$\begin{array}{c} p_{U}\left(u\right)=\int_{S}p_{Y}\left(g^{-1}\left(u,v;\alpha \right)\right)|\frac{\partial g_{1}}{\partial y_{1}}\left(g^{-1}\left(u,v;\alpha \right)\right){|}^{-1}dv, \end{array}$$

where *S* is the support set of the random variable V.

Since the random variable $X^{\prime }|x$ can be written as a sum of *U* and *Z*, we find the probability density function by convolution as follows:

(11) $$\begin{array}{c} p_{X^{\prime }|x}\left(x^{\prime }|x\right)=\int p_{U}\left(u\right)p_{Z}\left(x^{\prime }-u\right)du. \end{array}$$

Now, the conditional differential entropy h(Z|X,Y) is in terms of these probability densities. It is useful that translation does not change the differential entropy, $h_{\epsilon }\left(f\left(X,Y\right)+Z|X,Y\right)=h\left(Z|X,Y\right)$. In addition, *Z* is independent from X,Y, h(Z|X,Y)=h(Z). Now, we define

(12) $$\begin{array}{c} h\left(f\left(X,Y\right)|X,Y\right):=\lim_{\epsilon \to 0^{+}}h_{\epsilon }\left(f\left(X,Y\right)+Z|X,Y\right) \end{array}$$

if this limit exists.

We consider two scenarios: (1) *Z* is a uniform random variable or (2) *Z* is a Gaussian random variable. If it is uniform in the interval [−ϵ/2,ϵ/2], then the differential entropy is h(Z)=ln(ϵ). If specifically, *Z* is Gaussian with zero mean and ϵ standard deviation, then $h\left(Z\right)=\frac{1}{2}\ln\left(2\pi e\epsilon^{2}\right)$. Therefore, $h_{\epsilon }\left(f\left(X,Y\right)+Z|X,Y\right)\to -\infty$ as $\epsilon \to 0^{+}$ in both cases. Therefore, h(f(X,Y)|X,Y)) is not finite in this definition (Equation (12)) as well. Thus, instead of calculating $X^{\prime }=f\left(X,Y\right)$, we need to use a noisy version of data $X^{\prime }=f\left(X,Y\right)+Z$. For that case,

(13) $$\begin{array}{c} h\left(X^{\prime }|X,Y\right)=h\left(Z\right)=\left\{\begin{array}{cc} \ln(\epsilon ); & Z∼U(-\epsilon /2,\epsilon /2)\\ \frac{1}{2}\ln\left(2\pi e\epsilon^{2}\right); & Z∼N(0,\epsilon^{2}) \end{array}\right., \end{array}$$

where U(−ϵ/2,ϵ/2) is the uniform distribution in the interval [−ϵ/2,ϵ/2], and $N(0,\epsilon^{2})$ is a Gaussian distribution with zero mean and ϵ standard deviation.

Now, we focus on $h(X^{\prime }|X)$. If $X^{\prime }$ is just a function of *X*, then we can similarly show that: if $X^{\prime }=f\left(X\right),$ then

(14) $$\begin{array}{c} h\left(f\left(X\right)+Z|X\right)=h\left(Z\right)=\left\{\begin{array}{cc} \ln(\epsilon ); & Z∼U(-\epsilon /2,\epsilon /2)\\ \frac{1}{2}\ln\left(2\pi e\epsilon^{2}\right); & Z∼N(0,\epsilon^{2}). \end{array}\right. \end{array}$$

In addition, notice that, if $X^{\prime }=f\left(X,Y\right)$, then $h(X^{\prime }|X)$ will exist, and most of the cases will be finite. However, when we calculate $T_{y\to x}$, we need to use the noisy version to avoid the issues in calculating $h(X^{\prime }|X,Y)$. We will now consider the interesting case $X^{\prime }=f\left(X,Y\right)+Z$ and calculate $h(X^{\prime }|X)$. We require $p_{X^{\prime }|X}$ and Equation (11) can be used to calculate this probability. Let us denote $I:=\int p_{U}\left(u\right)p_{Z}\left(x^{\prime }-u\right)du;$ then,

(15) $$\begin{array}{cc} h_{\epsilon }\left(X^{\prime }|X\right) & =\int \int I\;p_{X}\left(x\right)\ln\left(I\right)dx^{\prime }dx\\ \; & =\int p_{X}\left(x\right)\int I\ln\left(I\right)dx^{\prime }dx\\ \; & =E_{X}\left(Q\right), \end{array}$$

where $Q=\int I\ln\left(I\right)dx^{\prime }$. Notice that, if *Q* does not depend on x, then $h\left(X^{\prime }|X\right)=Q\int p_{X}dx=Q$ because $\int p_{X}dx=1$(since $p_{x}$ is a probability density function). Therefore, we can calculate $h_{\epsilon }\left(X^{\prime }|X\right)$ by four steps. First, we calculate the density function for U=f(x,Y) (by using Equation (9) or (10)). Then, we calculate $I=p_{X^{\prime }|X}$ by using Equation (11). Next, we calculate the value of Q, and finally we calculate the value of $h_{\epsilon }\left(X^{\prime }|X\right)$.

Thus, the transfer entropy from *y* to *x* follows in terms of comparing conditional entropies,

(16) $$\begin{array}{c} T_{y\to x}=h\left(X^{\prime }|X\right)-h\left(X^{\prime }|X,Y\right). \end{array}$$

This quantity is not well defined when $X^{\prime }=f\left(X,Y\right)$, and therefore we considered the $X^{\prime }=f\left(X,Y\right)+Z$ case. This interpretation of transfer entropy depends on the parameter ϵ, as we define

(17) $$\begin{array}{c} T_{y\to x}:=\lim_{\epsilon \to 0^{+}}T_{y\to x}\left(\epsilon \right)=\lim_{\epsilon \to 0^{+}}h_{\epsilon }\left(X^{\prime }|X\right)-h_{\epsilon }\left(X^{\prime }|X,Y\right) \end{array}$$

if this limit exists.

Note that

(18) $$\begin{array}{c} T_{y\to x}=\left\{\begin{array}{cc} \lim_{\epsilon \to 0^{+}}h\left(Z\right)-h\left(Z\right)=0;\; & X^{\prime }=f\left(X\right)\\ \infty ;\; & X^{\prime }=f\left(X,Y\right)\neq f\left(X\right). \end{array}\right. \end{array}$$

Thus, we see that a finite quantity is ensured by the noise term. We can easily find an upper bound for the transfer entropy when $X^{\prime }=f\left(X,Y\right)+Z$ is a random variable with finite support (with all the other assumptions mentioned earlier) and suppose Z∼U(−ϵ/2,ϵ/2). First, notice that the uniform distribution maximizes entropy amongst all distributions of continuous random variables with finite support. If *f* is component-wise monotonically increasing continuous function, then the support of $X^{\prime }|x$ is $\left[f\left(x,y_{min}\right)-\epsilon /2,f\left(x,y_{min}\right)+\epsilon /2\right]$ for all $x\in \Omega_{x}$. Here, $y_{min}$ and $y_{max}$ are minimum and maximum values of *Y*. Then, it follows that

(19) $$\begin{array}{c} h_{\epsilon }\left(X^{\prime }|X\right)\leq ln(|f\left(x_{max},y_{max}\right)-f\left(x_{max},y_{min}\right)+\epsilon \left|\right), \end{array}$$

where $x_{max}$ is the maximum *x* value. We see that an interesting upper bound for transfer entropy follows:

(20) $$\begin{array}{c} T_{y\to x}\left(\epsilon \right)\leq \ln\left(|\frac{f\left(x_{max},y_{max}\right)-f\left(x_{max},y_{min}\right)}{\epsilon }+1|\right). \end{array}$$

### 2.3. Relating Transfer Entropy to a Geometric Bound

Noting that transfer entropy and other variations of the G-causality concept are expressed in terms of conditional probabilities, we recall that

(21) $$\rho \left(x^{\prime }|x,y\right)\rho (x,y)=\rho (x,y,x^{\prime }).$$

Again, we continue to overload the notation on the functions ρ, the details of the arguments distinguishing to which of these functions we refer.

Now, consider the change of random variable formulas that map between probability density functions by smooth transformations. In the case that $x^{\prime }=f_{1}\left(x\right)$ (in the special case that $f_{1}$ is one-one), then

(22) $$\rho \left(x^{\prime }\right)=\frac{\rho (x)}{\left|\frac{df_{1}}{dx}\left(x\right)\right|}=\frac{\rho (f_{1}^{-1}\left(x^{\prime }\right))}{|\frac{df_{1}}{dx}\left(f_{1}^{-1}\left(x^{\prime }\right)\right)|}.$$

In the more general case, not assuming one-one-ness, we get the usual Frobenius–Perron operator,

(23) $$\rho \left(x^{\prime }\right)=\sum_{x:x^{\prime }=f_{1}\left(x\right)}\rho \left(x,x^{\prime }\right)=\sum_{x:x^{\prime }=f_{1}\left(x\right)}\frac{\rho (x)}{\left|\frac{df_{1}}{dx}\left(x\right)\right|},$$

in terms of a summation over all pre-images of $x^{\prime }$. Notice also that the middle form is written as a marginalization across *x* of all those *x* that lead to $x^{\prime }$. This Frobenius–Perron operator, as usual, maps densities of ensembles of initial conditions under the action of the map $f_{1}$.

Comparing to the expression

(24) $$\rho \left(x,x^{\prime }\right)=\rho \left(x^{\prime }|x\right)\rho \left(x\right),$$

we assert the interpretation that

(25) $$\rho \left(x^{\prime }|x\right):=\frac{1}{|\frac{df_{1}}{dx}\left(x\right)|}\delta \left(x^{\prime }-f_{1}\left(x\right)\right),$$

where δ is the Dirac delta function. In the language of Bayesian uncertainty propagation, $p(x^{\prime }|x)$ describes the likelihood function, if interpreting the future state $x^{\prime }$ as data, and the past state *x* as parameters, in a standard Bayes description, p(data|parameter)×p(parameter). As usual for any likelihood function, while it is a probability distribution over the data argument, it may not necessarily be so with respect to the parameter argument.

Now, consider the case where $x^{\prime }$ is indeed nontrivially a function with respect to not just *x*, but also with respect to *y*. Then, we require the following asymmetric space transfer operator, which we name here an asymmetric Frobenius–Perron operator for smooth transformations between spaces of dissimilar dimensionality:

**Theorem** **1** (Asymmetric Space Transfer Operator). *If $x^{\prime }=f_{1}\left(x,y\right)$, for $f_{1}:\Omega_{1}\to \Upsilon$, given bounded open domain $\left(x,y\right)\in \Omega_{1}\subset R^{2d}$, and range $x^{\prime }\in \Upsilon \subset R^{d}$, and $f_{1}\in C^{1}\left(\Omega_{1}\right)$, and the Jacobian matrices, $\frac{\partial f_{1}}{\partial x}\left(x,y\right)$, and $\frac{\partial f_{1}}{\partial y}\left(x,y\right)$ are not both rank deficient at the same time, then taking the initial density $\rho \left(x,y\right)\in L^{1}\left(\Omega_{1}\right)$, the following serves as a transfer operator mapping asymmetrically defined densities $P:L^{1}\left(\Omega_{1}\right)\to L^{1}\left(\Upsilon \right)$*

(26) $$\rho \left(x^{\prime }\right)=\sum_{\left(x,y\right):x^{\prime }=f_{1}\left(x,y\right)}\rho \left(x,y,x^{\prime }\right)=\sum_{\left(x,y\right):x^{\prime }=f_{1}\left(x,y\right)}\frac{\rho (x,y)}{\left|\frac{\partial f_{1}}{\partial x}\left(x,y\right)\right|+\left|\frac{\partial f_{1}}{\partial y}\left(x,y\right)\right|}.$$

The proof of this is in Appendix A. Note also that, by similar argumentation, one can formulate the asymmetric Frobenius–Perron type operator between sets of dissimilar dimensionality in an integral form.

**Corollary** **1** (Asymmetric Transfer Operator, Kernel Integral Form). *Under the same hypothesis as Theorem 1, we may alternatively write the integral kernel form of the expression,*

(27) $$\begin{array}{cccc} P:L^{2}\left(R^{2}\right) & \to & L^{2}\left(R\right) & \\ \rho (x,y) & & \mapsto & \rho^{\prime }\left(x^{\prime }\right)=P\left[\rho \right]\left(x,y\right)]\\ & & = & \\ & & = & \int_{L_{x^{\prime }}}\rho \left(x,y,x^{\prime }\right)dxdy=\int_{L_{x^{\prime }}}\rho \left(x^{\prime }|x,y\right)\rho \left(x,y\right)dxdy \end{array}$$

(28) $$\begin{array}{cc} = & \int_{L_{x^{\prime }}}\frac{1}{|\frac{\partial f_{1}}{\partial x}\left(x,y\right)|+|\frac{\partial f_{1}}{\partial y}\left(x,y\right)|}\rho \left(x,y\right)dxdy. \end{array}$$

**Figure 4:**
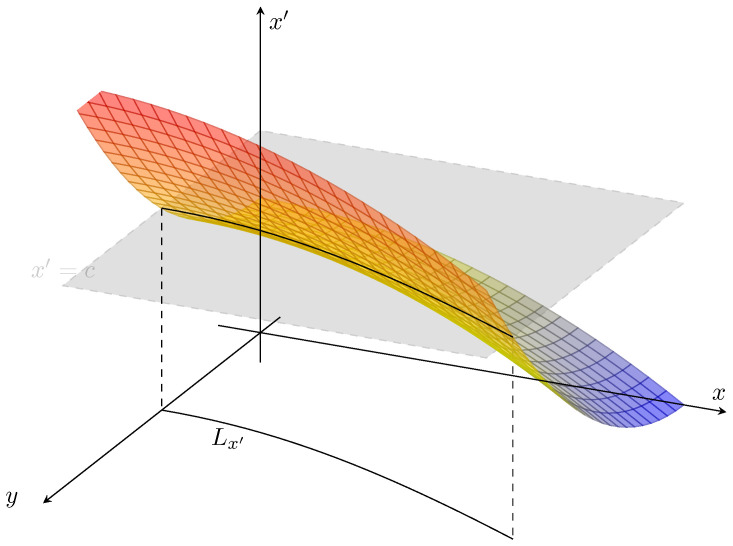
The asymmetric transfer operator, Equation (27), is written in terms of intefration over the level set, $L_{x^{\prime }}$ of $x^{\prime }=f_{1}\left(x,y\right)$ associated with a fixed value $x^{\prime }$, Equation (29).

*This is in terms of a line integration along the level set, $L_{x^{\prime }}$. See Figure 4:*

(29) $$L_{x^{\prime }}=\left\{\left(x,y\right)\in \Omega_{1}:f\left(x,y\right)=x^{\prime }a\;chosen\;constant.\right\}$$

In Figure 4, we have shown a typical scenario where a level set is a curve (or it may well be a union of disjoint curves), whereas, in a typical FP-operator between sets of the same dimensionality, generally the integration is between pre-images that are usually either singletons, or unions of such points, $\rho^{\prime }\left(x^{\prime }\right)=\int \delta \left(s-f\left(x\right)\right)\rho \left(s\right)ds=\sum_{x:f\left(x\right)=x^{\prime }}\frac{\rho (x)}{\left|Df(x)\right|}$.

Contrasting standard and the asymmetric forms of transfer operators as described above, in the next section, we will compute and bound estimates for the transfer entropy. However, it should already be apparent that, if $\frac{\partial f_{1}}{\partial y}=0$ in probability with respect to ρ(x,y), then $T_{y\to x}=0$.

**Comparison to other statistical divergences reveals geometric relevance:** Information flow is quite naturally defined by the KL-divergence, in that it comes in the units of entropy, e.g., bits per second. However, the well-known Pinsker’s inequality [25] allows us to more easily relate the transfer entropy to a quantity that has a geometric relevance using the total variation, even if this is only by an inequality estimate.

Recall that Pinsker’s inequality [25] relates random variables with probability distributions *p* and *q* over the same support to the total variation and the KL-divergence as follows:

(30) $$0\leq \frac{1}{2}TV\left(P,Q\right)\leq \sqrt{D_{KL}\left(P||Q\right)},$$

written as probability measures *P*, *Q*. The total variation distance between probability measures is a maximal absolute difference of possible events,

(31) $$TV\left(P,Q\right)=\underset{A}{\sup}\left|P\left(A\right)-Q\left(A\right)\right|,$$

but it is well known to be related to 1/2 of the L1-distance in the case of a common dominating measure, p(x)dμ=dP, q(x)dμ=dQ. In this work, we only need absolute continuity with respect to Lebesgue measure, p(x)=dP(x), q(x)=dQ(x); then,

(32) $$TV\left(P,Q\right)=\frac{1}{2}\int |p\left(x\right)-q\left(x\right)|dx=\frac{1}{2}{∥p-q∥}_{L^{1}},$$

here with respect to Lebesgue measure. In addition, we write $D_{KL}\left(P||Q\right)=\int p\left(x\right)\log\frac{p(x)}{q(x)}dx$; therefore,

(33) $$\frac{1}{2}{∥p-q∥}_{L^{1}}^{2}\leq \int p\left(x\right)\log\frac{p(x)}{q(x)}dx.$$

Thus, with the Pinsker inequality, we can bound the transfer entropy from below by inserting the definition Equation (3) into the above:

(34) $$0\leq \frac{1}{2}{∥p\left(x^{\prime }|x,y\right)-p\left(x^{\prime }|x\right)∥}_{L^{1}}^{2}\leq T_{y\to x}.$$

The assumption that the two distributions correspond to a common dominating measure requires that we interpret $p(x^{\prime }|x)$ as a distribution averaged across the same ρ(x,y) as $p(x^{\prime }|x,y)$. (Recall by definition [26] that λ is a common dominating measure of *P* and *Q* if p(x)=dP/dλ and q(x)=dQ/dλ describe corresponding densities). For the sake of simplification, we interpret transfer entropy relative to a uniform initial density, ρ(x,y), for both entropies of Equation (16). With this assumption, we interpret

(35) $$0\leq \frac{1}{2}{∥\frac{1}{|\frac{\partial f_{1}}{\partial x}\left(x,y\right)|+|\frac{\partial f_{1}}{\partial y}\left(x,y\right)|}-\frac{1}{\left|\frac{df_{1}}{dx}\left(x\right)\right|}∥}_{L^{1}\left(\Omega_{1},\rho \left(x,y\right)\right)}^{2}\leq T_{y\to x}.$$

In the special case that there is very little information flow, we would expect that $|\frac{\partial f_{1}}{\partial y}|<b<<1$, and $b<<|\frac{\partial f_{1}}{\partial x}|$, almost every x,y; then, a power series expansion in small *b* gives

(36) $$\frac{1}{2}{∥\frac{1}{\left|\frac{\partial f_{1}}{\partial x}\left(x,y\right)\right|+\left|\frac{\partial f_{1}}{\partial y}\left(x,y\right)\right|}-\frac{1}{\left|\frac{df_{1}}{dx}\left(x\right)\right|}∥}_{L^{1}\left(\Omega_{1},\rho \left(x,y\right)\right)}^{2}\approx \frac{Vol(\Omega_{1})}{2}\frac{<|\frac{\partial f_{1}}{\partial y}|{>}^{2}}{<|\frac{\partial f_{1}}{\partial x}|{>}^{4}},$$

which serves approximately as the TV-lower bound for transfer entropy where have used the notation <·> to denote an average across the domain. Notice that, therefore, $\delta (p\left(x^{\prime }|x,y\right),p\left(x^{\prime }|x\right))↓$ as $|\frac{\partial f_{1}}{\partial y}|↓$. While Pinsker’s inequality cannot guarantee that $T_{y\to x}↓$, since TV is only an upper bound, it is clearly suggestive. In summary, comparing inequality Equation (35) to the approximation (36) suggests that, for $|\frac{\partial f_{1}}{\partial y}\left|<<b<<\right|\frac{\partial f_{1}}{\partial x}|$, for b>0, for a.e. x,y, then $T_{y\to x}↓$ as b↓.

Now, we change to a more computational direction of this story of interpreting information flow in geometric terms. With the strong connection described in the following section, we bring to the problem of information flow between geometric concepts to information flow concepts, such as entropy, it is natural to turn to studying the dimensionality of the outcome spaces, as we will now develop.

Part II: Numerics and Examples of Geometric Interpretations 

Now, we will explore numerical estimation aspects of transfer entropy for causation inference in relationship to geometry as described theoretically in the previous section, and we will compare this numerical approach to geometric aspects.

## 3. Geometry of Information Flow

As theory suggests, see the sections above, there is a strong relationship between the information flow (causality as measured by transfer entropy) and the geometry, encoded for example in the estimates leading to Equation (36). The effective dimensionality of the underlying manifold as projected into the outcome space is a key factor to identify the causal inference between chosen variables. Indeed, any question of causality is in fact observer dependent. To this point, suppose $x^{\prime }$ only depends on x,y and $x^{\prime }=f\left(x,y\right)$, where $f\in C^{1}\left(\Omega_{1}\right)$. We noticed that (Section 2) $T_{y\to x}=0\Leftrightarrow \frac{\partial f}{\partial y}=0,\;\forall \left(x,y\right)\in \Omega_{1}$. Now, notice that $\frac{\partial f}{\partial y}=0,\;\forall \left(x,y\right)\in \Omega_{1}\Leftrightarrow x^{\prime }=f\left(x,y\right)=f\left(x\right).$ Therefore, in the case that $\Omega_{1}$ is two-dimensional, then $\left(x,x^{\prime }\right)$ would be a one-dimensional, manifold if and only if $\frac{\partial f}{\partial y}=0,\;\forall \left(x,y\right)\in \Omega_{1}$. See Figure 3. With these assumptions,

$$T_{y\to x}=0\Leftrightarrow \left(x,x^{\prime }\right)\;\mathrm{data}\;\mathrm{lie}\;\mathrm{on}\;a1D\;\mathrm{manifold}.$$

Likewise, for more general dimensionality of the initial $\Omega_{1}$, the story of the information flow between variables is in part a story of how the image manifold is projected. Therefore, our discussion will focus on estimating the dimensionality in order to identify the nature of the underlying manifold. Then, we will focus on identifying causality by estimating the dimension of the manifold, or even more generally of the resulting set if it is not a manifold but perhaps even a fractal. Finally, this naturally leads us to introduce a new geometric measure for characterizing the causation, which we will identify as $Geo_{y\to x}$.

### 3.1. Relating the Information Flow as Geometric Orientation of Data

For a given time series $x:=x_{n}\in R^{d_{1}},y:=y_{n}\in R^{d_{2}}$, consider the $x^{\prime }:=x_{n+1}$ and *contrast* the dimensionalities of $\left(x,y,x^{\prime }\right)$ versus $\left(x,x^{\prime }\right)$, in order to identify that $x^{\prime }=f\left(x\right)$ or $x^{\prime }=f\left(x,y\right)$. Thus, in mimicking the premise of Granger causality, or likewise of Transfer entropy, contrasting these two versions of the explanations of $x^{\prime }$, in terms of either (x,y) or *x*, we decide the causal inference, but this time, by using only the geometric interpretation. First, we recall how fractal dimensionality evolves under transformations, [27].

**Theorem** **2** ([27]). *Let A be a bounded Borel subset of $R^{d_{1}}$. Consider the function $F:A\to R^{d_{1}}\times R^{d_{1}}$ such that $F\left(x\right)=\left(x,x^{\prime }\right)$ for some $x^{\prime }\in R^{d_{1}}$. The correlation dimension $D_{2}\left(F\left(A\right)\right)\leq d_{1}$, if and only if there exists a function $f:A\to R^{d_{1}}$ such that $x^{\prime }=f\left(x\right)$ with $f\in C^{1}\left(A\right)$.*

The idea of the arguments in the complete proof found in Sauer et. al., [27], are as follows. Let *A* be bounded Borel subset of $R^{d_{1}}$ and $f:A\to R^{d_{1}}$ with $f\in C^{1}\left(A\right)$. Then, $D_{2}\left(f\left(A\right)\right)=D_{2}\left(A\right)$, where $D_{2}$ is the correlation dimension [28]. Note that $D_{2}\left(A\right)\leq d_{1}$. Therefore, $D_{2}\left(F\left(A\right)\right)=D_{2}\left(A\right)\leq d_{1}$, with $F:A\to R^{d_{1}}\times R^{d_{1}}$ if and only if F(x)=(x,f(x)).

Now, we can describe this dimensional statement in terms of our information flow causality discussion, to develop an alternative measure of inference between variables. Let $\left(x,x^{\prime }\right)\in \Omega_{2}\subset R^{2d_{1}}$ and $\left(x,y,x^{\prime }\right)\in \Omega_{3}\subset R^{2d_{1}+d_{2}}$. We assert that there is a causal inference from *y* to *x*, if $dim\left(\Omega_{2}\right)>d_{1}$ and $d_{1}<dim\left(\Omega_{3}\right)\leq d_{1}+d_{2}$, (Theorem 1). In this paper, we focus on time series $x_{n}\in R$ which might also depend on time series $y_{n}\in R$, and we will consider the geometric causation from *y* to *x*, for $\left(x,y\right)\in A\times B=\Omega_{1}\subset R^{2}$. We will denote geometric causation by $GeoC_{y\to x}$ and assume that A,B are Borel subsets of R. Correlation dimension is used to estimate the dimensionality. First, we identify the causality using the dimensionality of on $\left(x,x^{\prime }\right)$ and $\left(x,y,x^{\prime }\right)$. Say, for example, that $\left(x,x^{\prime }\right)\in \Omega_{2}\subset R^{2}$ and $\left(x,y,x^{\prime }\right)\in \Omega_{3}\subset R^{3}$; then, clearly we would enumerate a correlation dimension causal inference from *y* to *x*, if $dim(\Omega_{2})>1$ and $1<dim(\Omega_{3})\leq 2$ (Theorem 1).

### 3.2. Measure Causality by Correlation Dimension

As we have been discussing, the information flow of a dynamical system can be described geometrically by studying the sets (perhaps they are manifolds) $X\times X^{\prime }$ and $X\times Y\times X^{\prime }$. As we noticed in the last section, comparing the dimension of these sets can be interpreted as descriptive of information flow. Whether dimensionality be estimated from data or by a convenient fractal measure such as the correlation dimension ($D_{2}\left(.\right)$), there is an interpretation of information flow when contrasting $X\times X^{\prime }$ versus $X\times Y\times X^{\prime }$, in a spirit reminiscent of what is done with transfer entropy. However, these details are geometrically more to the point.

Here, we define $GeoC_{y\to x}$ (geometric information flow) by GeoC(.|.) as conditional correlation dimension.

**Definition** **1** (Conditional Correlation Dimensional Geometric Information Flow). *Let M be the manifold of data set $\left(X_{1},\;X_{2},\;\cdots ,\;X_{n},\;X^{\prime }\right)$ and let $\Omega_{1}$ be the data set $\left(X_{1},\;X_{2},\;\cdots ,\;X_{n}\right)$. Suppose that the $M,\;\Omega_{1}$ are bounded Borel sets. The quantity*

(37) $$\begin{array}{c} GeoC\left(X^{\prime }|X_{1},\;\cdots ,\;X_{n}\right):=D_{2}\left(M\right)-D_{2}\left(\Omega_{1}\right) \end{array}$$

*is defined as “Conditional Correlation Dimensional Geometric Information Flow". Here, $D_{2}\left(.\right)$ is the usual correlation dimension of the given set, [29,30,31].*

**Definition** **2** (Correlation Dimensional Geometric Information Flow). *Let $x:=x_{n},y=y_{n}\in R$ be two time series. The correlation dimensional geometric information flow from y to x as measured by the correlation dimension and denoted by $GeoC_{y\to x}$ is given by*

(38) $$\begin{array}{c} \;GeoC_{y\to x}:=GeoC\left(X^{\prime }|X\right)-GeoC\left(X^{\prime }|X,Y\right). \end{array}$$

A key observation is to notice that, if $X^{\prime }$ is a function of $\left(X_{1},\;X_{2},\;\cdots ,\;X_{n}\right)$, then $D_{2}\left(M\right)=D_{2}\left(\Omega_{1}\right)$; otherwise, $D_{2}\left(M\right)>D_{2}\left(\Omega_{1}\right)$ (Theorem 1). If *X* is not influenced by *y*, then $GeoC(X^{\prime }|X)=0,$$GeoC(X^{\prime }|X,Y)=0$ and therefore $GeoC_{y\to x}=0$. In addition, notice that $GeoC_{y\to x}\leq D_{2}\left(X\right)$, where $X=\{x_{n}|n=1,2,\cdots \}$. For example, if $x_{n}\in R$, then $GeoC_{y\to x}\leq 1$. Since we assume that influence of any time series $z_{n}\neq x_{n},y_{n}$ to $x_{n}$ is relatively small, we can conclude that $GeoC_{y\to x}\geq 0$, and, if $x^{\prime }=f\left(x,y\right)$, then $GeoC(X^{\prime }|X,Y)=0$. Additionally, the dimension ($GeoC(X^{\prime }|X)$) in the $\left(X,X^{\prime }\right)$ data scores how much additional (other than *X*) information is needed to describe the $X^{\prime }$ variable. Similarly, the dimension $GeoC(X^{\prime }|X,Y)$ in the $\left(X,Y,X^{\prime }\right)$ data describes how much additional (other than X,Y) information is needed to define $X^{\prime }$. However, when the number of data points N→∞, the value $GeoC_{y\to x}$ is not negative (equal to the dimension of *X* data). Thus, theoretically, GeoC identifies a causality in the geometric sense we have been describing.

## 4. Results and Discussion

Now, we present specific examples to contrast the transfer entropy with our proposed geometric measure to further highlight the role of geometry in such questions. Table 1 provides a summary of our numerical results. We use synthetic examples with known underlining dynamics to understand the accuracy of our model. Calculating transfer entropy has theoretical and numerical issues for those chosen examples while our geometric approach accurately identifies the causation. We use the correlation dimension of the data because data might be fractals. Using a Hénon map example, we demonstrate that fractal data will not affect our calculations. Furthermore, we use a real-world application that has a positive transfer entropy to explain our data-driven geometric method. Details of these examples can be found in the following subsections.

### 4.1. Transfer Entropy

**Figure 5:**
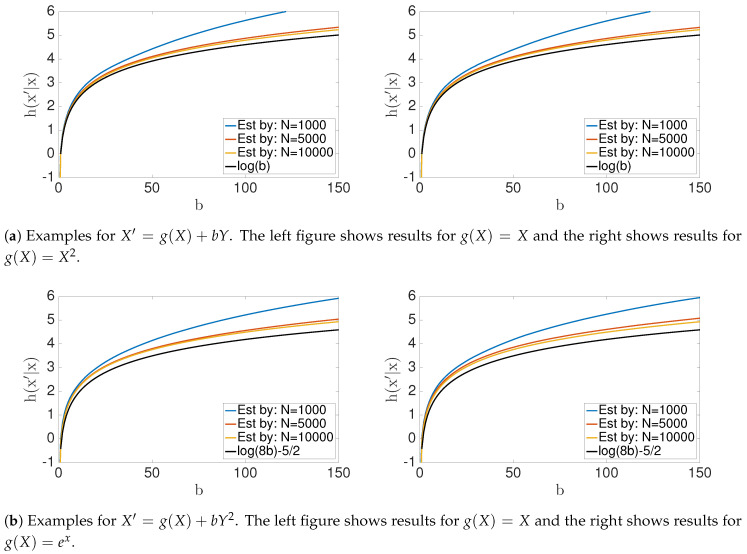
Conditional entropy *h*(*X*′|*X*). Note that these numerical estimates for the conditional entropy by the KSG method [32], converge (as *N* → ∞) to the analytic solutions (see Table 2).

**Figure 6:**
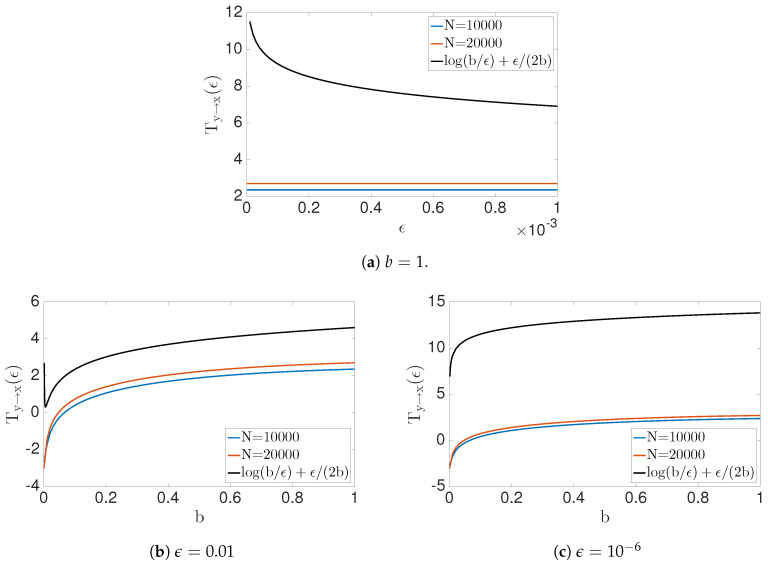
Numerical results and analytical results for transfer entropy *T*_*y*→*x*_(*ε*; *b*) to the problem *X*′ = *X* + *bY* + *ε*. Transfer entropy vs. *ε* shows in (**a**) for fixed *b* value. (**b**) and (**c**) show the behavior of the transfer entropy for *b* values with fixed e values. Notice that convergence of numerical solution is slow when epsilon is small.

In this section, we will focus on analytical results and numerical estimators for conditional entropy and transfer entropy for specific examples (see Figure 5 and Figure 6). As we discussed in previous sections starting with Section 2.2, computing the transfer entropy for $X^{\prime }=f\left(X,Y\right)$ has technical difficulties due to the singularity of the quantity $h(X^{\prime }|X,Y)$. First, we will consider the calculation of $h(X^{\prime }|X)$ for $X^{\prime }=f\left(X,Y\right)$, and then we will discuss the calculation for noisy data. In the following examples, we assumed that X,Y are random variables such that $X,Y\overset{iid}{∼}U\left(\left[1,2\right]\right)$. A summary of the calculations for a few examples are listed in Table 2.

We will discuss the transfer entropy with noisy data because making $h(X^{\prime }|X,Y)$ well defined requires absolute continuity of the probability density function $p(x,y,x^{\prime })$. Consider, for example, the problem form $X^{\prime }=g\left(X\right)+bY+C$, where X,Y are uniformly distributed independent random variables over the interval [1,2] (the same analysis can be extend to any finite interval) with *b* being a constant, and *g* a function of random variable *X*. We will also consider *C* to be a random variable, which is distributed uniformly on [−ϵ/2,ϵ/2]. Note that it follows that $h(X^{\prime }|X,Y)=\ln\epsilon$. To calculate the $h(X^{\prime }|X)$, we need to find the conditional probability $p(X^{\prime }|x)$ and observe that $X^{\prime }|x=U+C$, where U=g(x)+bY. Therefore,

(39) $$\begin{array}{c} p_{U}\left(u\right)=\left\{\begin{array}{cc} \frac{1}{b} & ;g_{1}\left(x\right)+b\leq X^{\prime }\leq g_{1}\left(x\right)+2b\\ 0 & ;otherwise. \end{array}\right. \end{array}$$

and

(40) $$\begin{array}{c} p_{X^{\prime }|X}\left(X^{\prime }|x\right)=\left\{\begin{array}{cc} \frac{x^{\prime }+\epsilon /2-g\left(x\right)}{b\epsilon } & ;g\left(x\right)-\epsilon /2\leq X^{\prime }\leq g\left(x\right)+\epsilon /2\\ \frac{1}{b} & ;g\left(x\right)+\epsilon /2\leq X^{\prime }\leq b+g\left(x\right)-\epsilon /2\\ \frac{-x^{\prime }+\epsilon /2+g\left(x\right)+b}{b\epsilon } & ;b+g\left(x\right)-\epsilon /2\leq X^{\prime }\leq b+g\left(x\right)+\epsilon /2\\ 0 & ;otherwise \end{array}\right.. \end{array}$$

By the definition of transfer entropy, we can show that

(41) $$\begin{array}{c} h\left(X^{\prime }|X\right)=\ln b+\frac{\epsilon }{2b} \end{array}$$

and hence transfer entropy of this data are given by

(42) $$\begin{array}{c} T_{y\to x}\left(\epsilon ;b\right)=\left\{\begin{array}{cc} \ln\frac{b}{\epsilon }+\frac{\epsilon }{2b}; & b\neq 0\\ 0; & b=0. \end{array}\right. \end{array}$$

Therefore, when b=0, the transfer entropy $T_{y\to x}=\ln\epsilon -\ln\epsilon =0$. In addition, notice that $T_{y\to x}\left(\epsilon ;b\right)\to \infty$ as ϵ→0. Therefore, convergence of the numerical estimates is slow when ϵ>0 is small (see Figure 6).

### 4.2. Geometric Information Flow

Now, we focus on quantifying the geometric information flow by comparing dimensionalities of the outcomes’ spaces. We will contrast this to the transfer entropy computations for a few examples of the form $X^{\prime }=g\left(X\right)+bY+C$.

**Figure 7:**
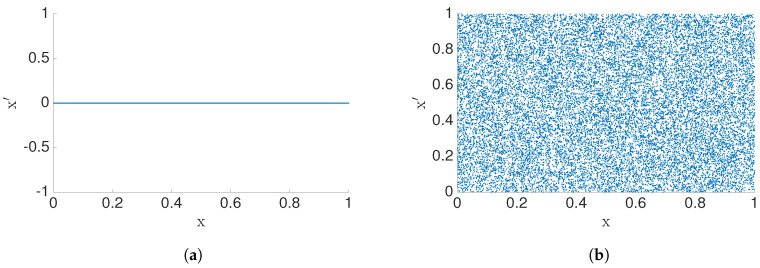
Manifold of the data (*x*′, *x*) with *x*′ = by and *y* is uniformly distributed in the interval [0, 1]. Notice that, when (**a**) *b* = 0, we have a 1D manifold, (**b**) *b* ≠ 0 we have 2D manifold, in the (*x*′, *x*) plane.

To illustrate the idea of geometric information flow, let us first consider a simple example, $x^{\prime }=ax+by+c$. If b=0, we have $x^{\prime }=f\left(x\right)$ and, when b≠0, we have the $x^{\prime }=f\left(x,y\right)$ case. Therefore, dimensionality of the data set $\left(x^{\prime },x\right)$ will change with parameter *b* (see Figure 7). When the number of data points N→∞ and b≠0, then $GeoC_{y\to x}\to 1$. Generally, this measure of causality depends on the value of *b*, but also the initial density of initial conditions.

**Figure 8:**
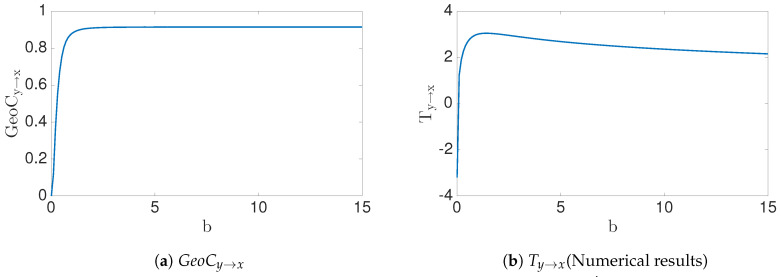
Geometric information flow vs. Transfer entropy for *X*′ = *bY* data.

In this example, we contrast theoretical solutions with the numerically estimated solutions (Figure 8). Theoretically, we expect $T_{y\to x}=\left\{\begin{array}{cc} 0 & ;b=0\\ \infty & ;b\neq 0 \end{array}\right.$ as N→∞. In addition, the transfer entropy for noisy data can be calculated by Equation (42).

### 4.3. Synthetic Data: $X^{\prime }=aX+bY$ with a≠0

The role of the initial density of points in the domain plays an important role in how the specific information flow values are computed depending on the measure used. To illustrate this point, consider the example of a unit square, ${\left[0,1\right]}^{2}$, that is uniformly sampled, and mapped by

(43) $$X^{\prime }=aX+bY,\;\mathrm{with}\;a\neq 0.$$

**Figure 9:**
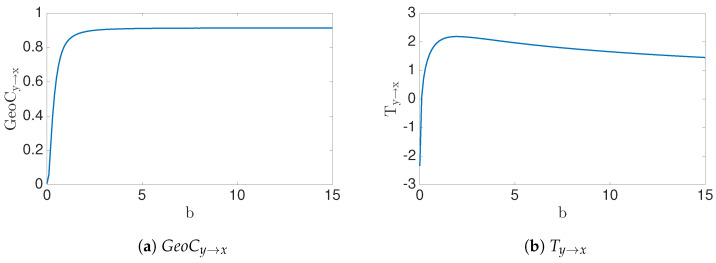
(**a**) shows the geometric information flow and (**b**) represents the Transfer entropy for *x*′ = *x* + *by* data. The figures show the changes with parameter *b*. We can notice that the transfer entropy has similar behavior to the geometric information flow of the data.

This fits our basic premise that $\left(x,y,x^{\prime }\right)$ data embeds in a 2D manifold, by ansatz of Equations (1) and (43), assuming for this example that each of x,y and $x^{\prime }$ are scalar. As the number of data point grows, N→∞, we can see that $GeoC_{y\to x}=\left\{\begin{array}{cc} 0 & ;b=0\\ 1 & ;b\neq 0 \end{array}\right.$ because $\left(X,X^{\prime }\right)$ data are on 2D manifold iff b≠0 (numerical estimation can be seen in Figure 9b). On the other hand, the conditional entropy $h(X^{\prime }|X,Y)$ is not defined, becoming unbounded when defined by noisy data. Thus, it follows that transfer entropy shares this same property. In other words, boundedness of transfer entropy depends highly on the $X^{\prime }|X,Y$ conditional data structure, while, instead, our geometric information flow measure highly depends on $X^{\prime }|X$ conditional data structure. Figure 9c demonstrates this observation with estimated transfer entropy and analytically computed values for noisy data. The slow convergence can be observed, Equation (42), Figure 6.

### 4.4. Synthetic Data: Nonlinear Cases

**Figure 10:**
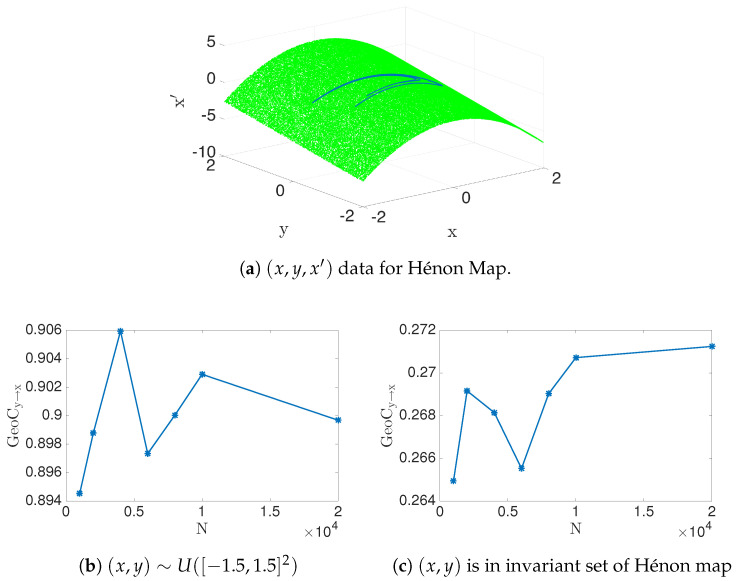
Consider the Hénon map, Equation (44), within the domain [−1.5, 1.5]^2^ and the invariant set of Hénon map. (**a**) the uniform distribution case (green) as well as the natural invariant measure of the attractor (blue) are shown regarding the (*x*, *y*, *x*′) data for both cases; (**b**) when (*x*, *y*) ∈ [−1.5, 1.5]^2^, notice that *GeoC*_*y*→*x*_ = 0.9, and (**c**) if (*x*, *y*) is in an invariant set of Hénon map, then *GeoC*_*y*→*x*_ = 0.2712.

Now, consider the Hénon map,

(44) $$\begin{array}{cc} x^{\prime } & =1-1.4x^{2}+y\\ y^{\prime } & =x \end{array}$$

as a special case of a general quadratic relationship, $x^{\prime }=ax+by^{2}+c$, for discussing how $x^{\prime }$ may depend on $\left(x,y\right)\in \Omega_{1}$. Again, we do not worry here if $y^{\prime }$ may or may not depend on *x* and or *y* when deciding dependencies for $x^{\prime }$. We will discuss two cases, depending on how the $\left(x,y\right)\in \Omega_{1}$ data are distributed. For the first case, assume (x,y) is uniformly distributed in the square, ${\left[-1.5,1.5\right]}^{2}$. The second and dynamically more realistic case will assume that (x,y) lies on the invariant set (the strange attractor) of the Hénon map. The geometric information flow is shown for both cases in Figure 10. We numerically estimate the transfer entropy for both cases, which gives $T_{y\to x}=2.4116$ and 0.7942, respectively. (However, recall that the first case for transfer entropy might not be finite analytically, and there is slow numerical estimation—see Table 3).

### 4.5. Application Data

**Figure 11:**
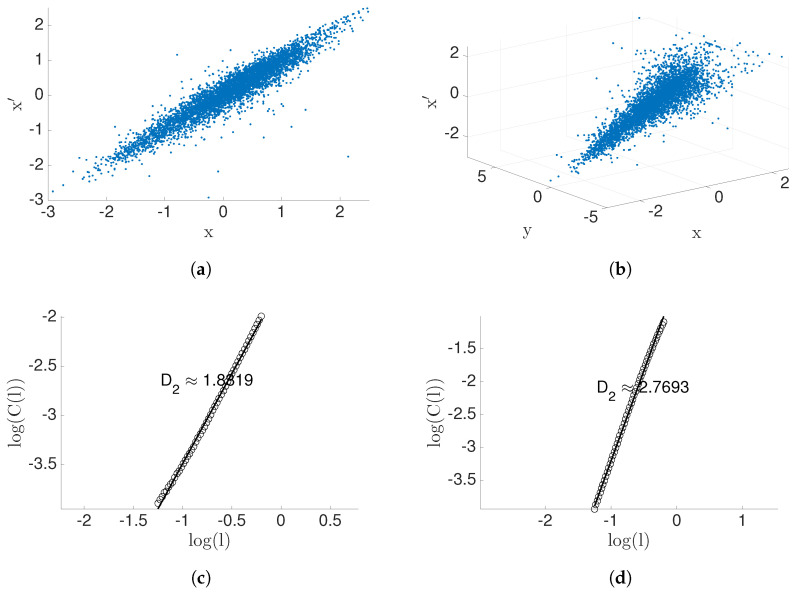
Result for heart rate(*x_n_*) (**a**,**c**) vs. breathing rate(*y_n_*) data (**b**,**d**). The top row is the scatter plot of the data, and the second row represents the dimension of the data.

Now, moving beyond bench-marking with synthetic data, we will contrast the two measures of information flow in a real world experimental data set. Consider heart rate ($x_{n}$) vs. breathing rate ($y_{n}$) data (Figure 11) as published in [33,34], consisting of 5000 samples. Correlation dimension of the data *X* is $D_{2}\left(X\right)=1.00$, and $D_{2}\left(X,X^{\prime }\right)=1.8319>D_{2}\left(X\right)$. Therefore, $X^{\prime }=X_{n+1}$ depends not only on *x*, but also on an extra variable (Theorem 2). In addition, correlation dimension of the data (X,Y) and $\left(X,Y,X^{\prime }\right)$ is computed $D_{2}\left(X,Y\right)=1.9801$ and $D_{2}\left(X,Y,X^{\prime }\right)=2.7693>D_{2}\left(X,Y\right)$, respectively. We conclude that $X^{\prime }$ depends on extra variable(s) other that (x,y) (Theorem 2) and the correlation dimension geometric information flow, $GeoC_{y\to x}=0.0427$, is computed by Equations (38) and (37). Therefore, this suggests the conclusion that there is a causal inference from breathing rate to heart rate. Since breathing rate and heart rate share the same units, the quantity measured by geometric information flow can be described without normalizing. Transfer entropy as estimated by the KSG method [32] with parameter k=30 is $T_{y\to x}=0.0485$, interestingly relatively close to the GeoC value. In summary, both measures for causality (GeoC,T) are either zero or positive together. It follows that there exists a causal inference (see Table 4).

## 5. Conclusions

We have developed here a geometric interpretation of information flow as a causal inference as usually measured by a positive transfer entropy, $T_{y\to x}$. Our interpretation relates the dimensionality of an underlying manifold as projected into the outcome space and summarizes the information flow. Furthermore, the analysis behind our interpretation involves standard Pinsker’s inequality that estimates entropy in terms of total variation, and, through this method, we can interpret the production of information flow in terms of details of the derivatives describing relative orientation of the manifolds describing inputs and outputs (under certain simple assumptions).

A geometric description of causality allows for new and efficient computational methods for causality inference. Furthermore, this geometric perspective provides a different view of the problem and facilitates the richer understanding that complements the probabilistic descriptions. Causal inference is weaved strongly throughout many fields and the use of transfer entropy has been a popular black box tool for this endeavor. Our method can be used to reveal more details of the underling geometry of the data-set and provide a clear view of the causal inference. In addition, one can use the hybrid method of this geometric aspect and existing other methods in their applications.

We provided a theoretical explanation (part I: Mathematical proof of the geometric view of the problem) and numerical evidence (part 2: A data-driven approach for mathematical framework) of a geometric view for the causal inference. Our experiments are based on synthetic (toy problems) and practical data. In the case of synthetic data, the underlining dynamics of the data and the actual solution to the problem are known. For each of these toy problems, we consider a lot of cases by setting a few parameters. Our newly designed geometric approach can successfully capture these cases. One major problem may be if data describes a chaotic attractor. We prove theoretically (Theorem 2) and experimentally (by Hénon map example: in this toy problem, we also know actual causality) that correlation dimension serves to overcome this issue. Furthermore, we present a practical example based on heart rate vs. breathing rate variability, which was already shown to have positive transfer entropy, and here we relate this to show positive geometric causality.

Furthermore, we have pointed out that transfer entropy has analytic convergence issues when future data ($X^{\prime }$) are exactly a function of current input data (X,Y) versus more generally $\left(X,Y,X^{\prime }\right)$. Therefore, referring to how the geometry of the data can be used to identify the causation of the time series data, we develop a new causality measurement based on a fractal measurement comparing inputs and outputs. Specifically, the correlation dimension is a useful and efficient way to define what we call correlation dimensional geometric information flow, $GeoC_{y\to x}$. The $GeoC_{y\to x}$ offers a strongly geometric interpretable result as a global picture of the information flow. We demonstrate the natural benefits of $GeoC_{y\to x}$ versus $T_{y\to x}$, in several synthetic examples where we can specifically control the geometric details, and then with a physiological example using heart and breathing data.

## Figures and Tables

**Table 1:** Summary of the results. Here, we experiment our new approach by synthetics and real world application data.

Data	Transfer Entropy (Section 4.1)	Geometric Approach
Synthetic: f(x,y)=aX+bY+C, a,b,c∈R	Theoretical issues can be noticed. Numerical estimation have boundedness issues when b<<1.	Successfully identify the causation for all the cases (100%).
Synthetic: f(x,y)=$ag_{1}\left(X\right)+bg_{2}\left(Y\right)+C$, a,b,c∈R	Theoretical issues can be noticed. Numerical estimation have boundedness issues when b<<1.	Successfully identify the causation for all the cases (100%).
Hénon map: use data set invariant under the map.	special case of $aX^{2}+bY+C$ with a=−1.4,b=c=1. Estimated transfer entropy is positive.	Successfully identify the causation.
Application: heart rate vs. breathing rate	Positive transfer entropy.	Identify positive causation. It also provides more details about the data.

**Table 2:** Conditional entropy $h(X^{\prime }|X)$ for $X^{\prime }=f\left(X,Y\right)$, for specific parametric examples listed, under the assumption that $X,Y\overset{iid}{∼}U\left(\left[1,2\right]\right)$.

f(X,Y)	$h(X^{\prime }|X)$
g(X)+bY	ln(b)
$g\left(X\right)+bY^{2}$	ln(8b)−5/2
g(X)+bln(Y)	$\ln(\frac{b\;e}{4})$

**Table 3:** Hénon Map Results. Contrasting geometric information flow versus transfer entropy in two different cases, 1st relative to uniform distribution of initial conditions (reset each time) and 2nd relative to the natural invariant measure (more realistic).

Domain	GeoC	$T_{y\to x}$
${\left[-1.5,1.5\right]}^{2}$	0.90	2.4116
Invariant Set	0.2712	0.7942

**Table 4:** Heart rate vs. breathing rate data—contrasting geometric information flow versus transfer entropy in breath rate to heart rate.

${\mathrm{GeoC}}_{y\to x}$	$T_{y\to x}$
0.0427	0.0485

## Appendix A. On the Asymmetric Spaces Transfer Operators

In this section we prove Theorem 1 concerning a transfer operator for smooth transformations between sets of perhaps dissimilar dimensionality. In general, the marginal probability density can be found by integrating (or summation in the case of a discrete random variable) to marginalize the joint probability densities. When $x^{\prime }=f\left(x,y\right)$, the joint density $\left(x,y,x^{\prime }\right)$ is non-zero only at points on $x^{\prime }=f\left(x,y\right)$. Therefore, $\rho \left(x^{\prime }\right)=\sum_{\left(x,y\right):x^{\prime }=f\left(x,y\right)}\rho \left(x,y,x^{\prime }\right)$ and notice that $\rho \left(x,y,x^{\prime }\right)=\rho \left(x^{\prime }|x,y\right)\rho \left(x,y\right)$ (By Bayes theorem). Hence, $\rho \left(x^{\prime }\right)=\sum_{\left(x,y\right):x^{\prime }=f\left(x,y\right)}\rho \left(x^{\prime }|x,y\right)\rho \left(x,y\right)$ and we only need to show the following claims. We will discuss this by two cases. First, we consider $x^{\prime }=f\left(x\right)$ and then we consider more general case x=f(x,y). In higher dimensions we can consider similar scenarios of input and output variables, and correspondingly the trapezoidal bounding regions would need to be specified in which we can analytically control the variables.

**Proposition** **A1** (Claim). *Let X∈R be a random variable with probability density function ρ(x). Suppose ρ(x),ρ(.|x) are Radon–Nikodym derivatives (of induced measure with respect to some base measure μ) which is bounded above and bounded away from zero. In addition, let $x^{\prime }=f\left(x\right)$ for some function $f\in C^{1}\left(R\right)$. Then,*

$$\rho \left(x^{\prime }|X=x_{0}\right)=\lim_{\epsilon \to 0}d_{\epsilon }\left(x^{\prime }-f\left(x_{0}\right)\right)$$

*where $d_{\epsilon }\left(x^{\prime }-f\left(x_{0}\right)\right)=\left\{\begin{array}{cc} \frac{1}{2\epsilon |f^{\prime }\left(x_{0}\right)|} & ;|x^{\prime }-f\left(x_{0}\right)\left|<\epsilon \right|f^{\prime }\left(x_{0}\right)|\\ 0 & ;\;\mathrm{otherwise} \end{array}\right..$*

**Proof.** Let 1>>ϵ>0 and $x\in I_{\epsilon }=\left(x_{0}-\epsilon ,x_{0}+\epsilon \right)$. Since ρ is a Radon–Nikodym derivative with bounded above and bounded away from zero, $\rho \left(I_{\epsilon }\right)=\int_{I_{\epsilon }}\frac{d\rho }{d\mu }d\mu \geq \frac{m}{2\epsilon }$ where *m* is the infimum of the Radon–Nikodym derivative. Similarly $\rho \left(I_{\epsilon }\right)\leq \frac{M}{2\epsilon }$ where *M* is the supremum of the Radon–Nikodym derivative. In addition, $|x^{\prime }-f\left(x_{0}\right)\left|\approx \right|f^{\prime }\left(x_{0}\right)||x-x_{0}|$ for $x\in I_{\epsilon }$. Therefore, $x^{\prime }\in (f\left(x_{0}\right)-\epsilon |f^{\prime }\left(x_{0}\right)|,f\left(x_{0}\right)+\epsilon |f^{\prime }\left(x_{0}\right)\left|\right)=I_{\epsilon }^{\prime }$ when $x\in I_{\epsilon }$. Hence, $\rho \left(x^{\prime }|x\in I_{\epsilon }\right)=\rho \left(x^{\prime }\in I_{\epsilon }^{\prime }\right)$ and $\frac{m}{2\epsilon |f^{\prime }\left(x_{0}\right)|}\leq \rho \left(x^{\prime }|x\in I_{\epsilon }\right)\leq \frac{M}{2\epsilon |f^{\prime }\left(x_{0}\right)|}$. Therefore, $\rho \left(x^{\prime }|X=x_{0}\right)=\lim_{\epsilon \to 0}d_{\epsilon }\left(x^{\prime }-f\left(x_{0}\right)\right)$ □

**Proposition** **A2** (Claim). *2 Let X,Y∈R be random variables with joint probability density function ρ(x,y). Suppose ρ(x,y) and ρ(.|x,y) are Radon–Nikodym derivatives (of induced measure with respect to some base measure μ) which is bounded above and bounded away from zero. In addition, let $x^{\prime }=f\left(x,y\right)\in R$ for some function $f\in C^{1}\left(R\right)$. Then,*

$$\rho \left(x^{\prime }|X=x_{0},Y=y_{0}\right)=\lim_{\epsilon \to 0}d_{\epsilon }\left(x^{\prime }-f\left(x_{0},y_{0}\right)\right)$$

*where $d_{\epsilon }\left(x^{\prime }-f\left(x_{0},y_{0}\right)\right)=\left\{\begin{array}{cc} \frac{1}{2\epsilon (|f_{x}\left(x_{0},y_{0}\right)|+|f_{y}\left(x_{0},y_{0}\right)|)} & ;|x^{\prime }-f\left(x_{0},y_{0}\right)\left|<\epsilon (\right|f_{x}\left(x_{0},y_{0}\right)\left|+\right|f_{y}\left(x_{0},y_{0}\right)\left|\right)\\ 0 & ;\;\mathrm{otherwise} \end{array}\right..$*

**Proof.** Let 1>>ϵ>0 and $A_{\epsilon }=\left\{\left(x,y\right)\right|x\in \left(x_{0}-\epsilon ,x_{0}+\epsilon \right),y\in \left(y_{0}-\epsilon ,y_{0}+\epsilon \right)$. Since ρ is a Radon–Nikodym derivative with bounded above and bounded away from zero, $\rho \left(A_{\epsilon }\right)=\int_{A_{\epsilon }}\frac{d\rho }{d\mu }d\mu \geq \frac{m}{4\epsilon^{2}}$ where *m* is the infimum of the Radon–Nikodym derivative. Similarly, $\rho \left(A_{\epsilon }\right)\leq \frac{M}{4\epsilon^{2}}$ where *M* is the supremum of the Radon–Nikodym derivative. In addition, $|x^{\prime }-f\left(x_{0},y_{0}\right)\left|\approx \right|f_{x}\left(x_{0},y_{0}\right)||x-x_{0}\left|+\right|f_{y}\left(x_{0},y_{0}\right)||y-y_{0}|$ for $\left(x,y\right)\in A_{\epsilon }$. Therefore, $x^{\prime }\in (f\left(x_{0},y_{0}\right)-\epsilon (|f_{x}\left(x_{0},y_{0}\right)\left|+\right|f_{y}\left(x_{0},y_{0}\right)|),f\left(x_{0},y_{0}\right)+\epsilon (|f_{x}\left(x_{0},y_{0}\right)\left|+\right|f_{y}\left(x_{0},y_{0}\right)\left|)\right)=I_{\epsilon }^{\prime }$ when $\left(x,y\right)\in A_{\epsilon }$. Hence, $\rho \left(x^{\prime }|\left(x,y\right)\in A_{\epsilon }\right)=\rho \left(x^{\prime }\in I_{\epsilon }^{\prime }\right)$ and $\frac{m}{2\epsilon (|f_{x}\left(x_{0},y_{0}\right)|+|f_{y}\left(x_{0},y_{0}\right)|)}\leq \rho \left(x^{\prime }|x\in I_{\epsilon }\right)\leq \frac{M}{2\epsilon (|f_{x}\left(x_{0},y_{0}\right)|+|f_{y}\left(x_{0},y_{0}\right)|)}$. Therefore, $\rho \left(x^{\prime }|X=x_{0},Y=y_{0}\right)=\lim_{\epsilon \to 0}d_{\epsilon }\left(x^{\prime }-f\left(x_{0},y_{0}\right)\right)$. □

If *f* only depends on *x*, then the partial derivative of *f* with respect to *y* is equal to zero and which leads to the same result as clam 1.
